# Establishment of immortalized Egyptian Rousettus bat cell lines

**DOI:** 10.1002/2211-5463.13781

**Published:** 2024-02-19

**Authors:** Lanlan Bai, Tetsuya Tani, Takeshi Kobayashi, Ryotaro Nouda, Yuta Kanai, Yusuke Sano, Kazutoshi Takami, Hiroshi Tomita, Eriko Sugano, Taku Ozaki, Tohru Kiyono, Tomokazu Fukuda

**Affiliations:** ^1^ Graduate School of Science and Engineering Iwate University Japan; ^2^ Laboratory of Animal Reproduction, Department of Agriculture Kindai University Nara Japan; ^3^ Department of Virology, Research Institute for Microbial Diseases Osaka University Japan; ^4^ Local Independent Administrative Agency Tennoji Zoological Gardens Osaka Japan; ^5^ Osaka Municipal Tennoji Zoological Gardens Japan; ^6^ Exploratory Oncology Research & Clinical Trial Center National Cancer Center Chiba Japan; ^7^ Present address: *Toyohashi Zoo and Botanical Park Toyohashi Japan

**Keywords:** cell cycle regulator, Egyptian Rousettus bat, immortalized cells, SV40 large T antigen, TERT

## Abstract

The Egyptian Rousettus bat (*Rousettus aegyptiacus*) is a common fruit bat species that is distributed mainly in Africa and the Middle East. Bats serve as reservoir hosts for numerous pathogens. Human activities, such as hunting bats for food, managing vermin, and causing habitat loss, elevate the likelihood of transmission of bat pathogens to humans and other animals. Consequently, bat cell lines play a crucial role as research materials for investigating viral pathogens. However, the inherent limitation of finite cell division in primary cells necessitates the use of immortalized cells derived from various bat tissues. Herein, we successfully established six fibroblast cell lines derived from an infant bat heart and lungs and an elderly bat heart. Three of the six cell lines, called K4DT cells, were transduced by a combination of cell cycle regulators, mutant cyclin‐dependent kinase 4, cyclin D1, and human telomerase reverse transcriptase. The other three cell lines, named SV40 cells, were transfected with simian virus 40 large T antigen. Transgene protein expression was detected in the transduced cells. All three K4DT cell lines and one lung‐derived SV40 cell line were virtually immortalized and nearly maintained the normal diploid karyotypes. However, the two other heart‐derived SV40 cell lines had aberrant karyotypes and the young bat‐derived cell line stopped proliferating at approximately 40 population doublings. These bat cell lines are valuable for studying pathogen genomics and biology.

## Graphical abstract

Egyptian Rousettus bats serve as a reservoir for pathogens. Fibroblast cell lines K4DT and SV40 were established to overcome the limited cell division of primary bat cells. While three K4DT and one SV40 cell lines were virtually immortalized with normal karyotypes, two SV40 lines had aberrant karyotypes. These bat cell lines are valuable tools for the study of viral pathogens.

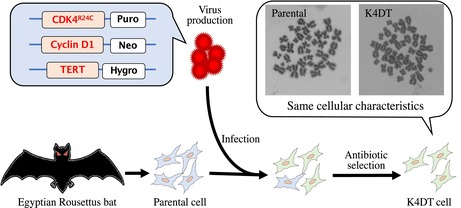

**Abbreviations**
ALT: alternative lengthening of telomeres
CDK4: cyclin‐dependent kinase 4
E2F: E2 promoter binding factor
IUCN: International Union for Conservation of Nature
PD: population doubling
pRB: retinoblastoma protein
SA‐β‐Gal: senescence‐associated beta‐galactosidase
SV40: simian virus 40
TERT: telomerase reverse transcriptase
TSCII: tuberous sclerosis type II gene

Bats are the second most diverse mammals, with 1456 reported species (https://batnames.org/) [1], and the only mammals capable of flying [2]. Bats are reservoir hosts for many viral, bacterial, and parasitic pathogens, often with little or no significant clinical signs of infection [3, 4]. Asymptomatic infections of particular pathogens in bats that are highly virulent in human hosts are of interest [4]. Bats are valuable animals for studying human pathogens because of their unique immune systems [3, 5]. Understanding how bat reservoir hosts respond to viral pathogen infections is essential for developing novel therapeutics and prevention measures for severely diseased hosts. Compared with bat colonies, cell lines are more convenient research materials because they are easy to manage, highly replicable, inexpensive, and have a low risk of viral infection. However, animal‐derived primary cells undergo a finite number of cell divisions and enter a non‐dividing state called senescence due to cultured stress or telomerase deficiency [5, 6, 7]. Cell lines can divide and proliferate indefinitely in culture, providing a consistent and uniform cell population useful for large‐scale studies, high‐throughput screening, and experiments requiring large numbers of cells. Therefore, bat cell lines are important tools for studying host–pathogen interactions, bat immunology, and zoonotic virus transmission. To date, lung epithelium‐like cell line, namely from *Tadarida brasiliensis* (Tb1Lu), *Rousettus aegyptiacus* immortalized fetal cell lines such as R06E and R05T, derived from fetal body and head, respectively [8], are commercially available. Various pathogens have evolved to infect specific tissues within the human body [9]. The choice of a specific cell line for studying a particular pathogen is crucial because different cell lines mimic the natural environment of the tissues they represent [10]. Further specification of bat cell culture models is required to specifically address different viral pathogenic processes in the reservoir host. The importance of selecting appropriate cell lines based on the target tissue to enhance the relevance and reliability of experimental results [10, 11]. In this study, we selected a megabat species, namely the Egyptian Rousettus bat (*Rousettus aegyptiacus*) [12], that is widely distributed across Africa, the Middle East, and India to provide new cell lines generated from lung and heart tissue for further studies on infectious diseases.

Cell proliferation occurs through the G1, S, G2, and M phases of the cell cycle, during which a cell divides into two sister cells [13]. In the G1 phase, transcriptional activator E2Fs are held inactive through their association with retinoblastoma protein (pRB), whereas in the G1/S phase, growth signals inactivate pRB function through cyclin‐dependent kinase‐mediated phosphorylation, thus releasing active E2F1 to activate genes required for the S phase. The cyclin‐dependent kinase inhibitor p16 prevents cells from entering the S phase by binding and inactivating CDK4 which, when active, phosphorylates pRB by forming a complex with cyclin D1 [14]. The mutant CDK4, CDK4^R24C^, which is originally found in melanoma cells, avoids binding with p16 and cannot be inactivated by p16. Telomeric DNA shortens at every cell division stage until it reaches a critical length. If it becomes too short, the cell cannot replicate because DNA damage signals are triggered and the cell cycle is arrested [15, 16]. In most cancers, human telomerase reverse transcriptase (TERT) is expressed, and its telomerase is positive [17]. In our previous studies, most animal‐derived primary cells were immortalized by expressing the R24C mutant of CDK4 (CDK4^R24C^), cyclin D1, and TERT [18, 19, 20]; however, African savannah elephant cells and chicken cells were not [21, 22].

The p53 gene is a tumor suppressor [23]; therefore, direct or indirect interference with its functions can lead to the loss of the cell cycle checkpoint. Furthermore, some DNA tumor virus‐encoded proteins inhibit p53 activity in repairing DNA damage. One of these proteins, simian virus 40 (SV40), a large tumor antigen, forms a complex with pRB and with p53, thereby inhibiting their functions [24, 25]. Therefore, SV40 large T antigen expression has been widely used to immortalize animal cells [26, 27] and has become a traditional immortalization method.

In this study, we attempted to immortalize cells derived from Egyptian Rousettus bats by expressing cell cycle regulators (CDK4^R24C^ and cyclin D1) together with TERT or the SV40 large T antigen. To ensure reproducibility, three lines of primary fibroblastic cells derived from an infant bat heart and lung and an elderly bat hearts were infected with enriched recombinant retroviruses expressing the cell cycle regulators and TERT or those expressing SV40. The cell cycle status, senescence, and karyotypes of the six established cell lines and parental cells were analyzed.

## Materials and methods

### Virus preparation

The viral packaging plasmids, pCL‐gag‐pol and VSVG‐RSV‐Rev, which were obtained from Dr. Hiroyuki Miyoshi (RIKEN BioResource Center, Tsukuba, Japan), were used for recombinant virus production. Briefly, packaging plasmids were co‐transfected with pQCXIP‐CDK4R24C, pQCXIN‐cyclin D1, pCLXSH‐TERT, pQCXIN‐SV40, or pQCXIN‐EGFP into 293T cells using polyethyleneimine‐max. After 72 h of transfection, the 293T culture medium was replaced with fresh medium supplemented with 10 μm forskolin and incubated for 48 h. Then, 30 mL of culture supernatants containing each recombinant virus were collected, and filtered through a 0.45‐μm filter (Sartorius AG, Göttingen, Germany), and 320 mg·mL^−1^ of PEG6000 was added at a ratio of 3 : 1 to concentrate at 2190 ***g*** for 60 min at 4 °C. The supernatant was discarded and the pellet was resuspended in 1.5 mL of fresh medium containing 4 μg·mL^−1^ of polybrene for infection.

### Cell immortalization

Three types of primary cells were derived from the fibroblasts of two individual bats that died at the Osaka Municipal Tennoji Zoological Gardens in Japan. Two of these came from the lungs and heart of an infant < 10 months old, whereas the other came from the heart of a 14 year old bat that had died of pneumonia (Table 1). The detailed information on bats has been described in the Ethical Statement. Primary cells were cultured in D‐MEM/Ham's F‐12 medium (FujiFilm Wako Pure Chemical Co., Osaka, Japan) containing 10% fetal bovine serum and 1× antibiotic mixture (Sigma‐Aldrich, St. Louis, MO, USA) and seeded at a density of 1 × 10^5^ cells per well in a collagen‐coated 6‐well plate. Concentrated recombinant viruses containing 4 μg·mL^−1^ of polybrene were inoculated to the pre‐cultured primary cells. After 96 h of inoculation, efficient infection with EGFP‐expressing retroviruses was confirmed by fluorescence microscopy. The transduced cells were named K4D and SV40, based on their introduced genes: K4D introduced the cassettes of the CDK4 mutant and cyclin D1 and SV40 introduced the cassettes of SV40. As a second step, the transduced cells were selected in the medium supplemented with 250–1000 μg·mL^−1^ of G418 and 250–500 ng·mL^−1^ of puromycin and then re‐infected with recombinant viruses containing TERT. The cells were then selected in medium supplemented with 4 mg·mL^−1^ of hygromycin B to obtain triply transduced cells and designated as K4DT cells.

**Table 1:** The summary of bat samples and their transduced cells.

Bat clone	Date of birth	Date of death	Age	Transduced cells	PD value^a^	Immortalization	Karyotype^b^
Infant bat	Jan 2018	25 Oct 2018	< 10 months old	Heart_K4DT	133	Success	Normal
				Heart_SV40	39	Failure	Abnormal
				Lung_K4DT	146	Success	Normal
				Lung_SV40	109	Success	Normal
Old bat	Autumn 2004	25 Dec 2018	14 years old	Heart‐2_K4DT	102	Success	Normal
				Heart‐2_SV40	111	Success	Abnormal

^a^ A population doubling (PD) value above 100 is considered an arbitrary threshold for cellular immortalization.

^b^ Karyotype of transduced cells compared to that of parental cells. If the chromosome expression pattern of the transduced cell was similar to that of the parental cells, it was defined as normal, otherwise it was defined as abnormal.

### Cell population doubling

The parental and transduced cells were seeded at a density of 4 × 10^4^ cells (initial number of cells, *N*_0_) per well in a 6‐well plate. While one type of cultured cells reached confluence, all types of culture cells were harvested, counted (final number of cells, *N*_f_), and serially passaged. The cell population doubling (PD) was calculated as the sum of $\log_{2}^{N_{f}/N_{0}}$.

### PCR and western blot

Genomic DNAs were extracted from parental and transduced cells at passage three using the NucleoSpin Tissue kit (Takara Bio, Shiga, Japan) according to the manufacturer's instructions. Genomic DNAs were subjected to genomic PCR by using KOD FX Neo (TOYOBO, Osaka, Japan) to amplify the introduced gene cassettes as well as the bat endogenous tuberous sclerosis type II gene (TSCII) as a positive control using the following primer sets; CDK4^R24C^, TF1066_5′‐CTTCCCATCAGCACAGTTCGTGAGG‐3′ and TF1067_5′‐AAAGATTTTGCCCAACTGGTCGGCTTC‐3′; cyclin D1, TF1064_5′‐CCCGATGCCAACCTCCTCAACGAC‐3′ and TF1065‐5′‐ATGATCTGTTTGTTCTCCTCCGCCTCTG‐3′; TERT, TF961_5′‐CTGCTCCTGCGTTTGGTGGATGATT‐3′ and TF963_5′‐GTCCTGAGTGACCCCAGGAGTGGCA‐3′; SV40, TF1068_5′‐ATGTATAGTGCCTTGACTAGAGATCCAA‐3′ and TF1069_5′‐CCAGCCATCCATTCTTCTATGTC‐3′; TSCII, TF959_5′‐CAGACCCTGCAGGACATTCTTG‐3′ and TF960_5′‐AGTGTCCAGGAACTCCAGCAA‐3′.

Each type of cell lysate was extracted with homogenization buffer (50 mm Tris–HCl pH 7.3, 150 mm NaCl, 1% Triton X‐100, 2.5 mg·mL^−1^ sodium deoxycholate and proteinase inhibitor) by sonication at 40 Hz. The protein concentration of the cell lysates was quantified using a bicinchoninic acid protein kit (Thermo Fisher Scientific, Waltham, MA, USA). Aliquots containing 12 μg of total cell lysates for each sample were separated by 10% sodium dodecyl sulphate‐polyacrylamide gel electrophoresis (SDS/PAGE). After blocking with 3% skim milk in phosphate buffer saline (PBS)‐T, the following primary antibodies were used: mouse anti‐CDK4 monoclonal (Santa Cruz Biotechnology, Dallas, TX, USA; code no. sc‐56277, 1 : 2000 dilution), rabbit anti‐cyclin D1 monoclonal (Medical & Biological Laboratories Co., Ltd., Aichi, Japan, code no. 553, 1 : 5000 dilution), mouse anti‐ SV40 monoclonal (Medical & Biological Laboratories Co., Ltd.; code no. 147, 1 : 2000 dilution) and anti‐α‐tubulin monoclonal (Santa Cruz Biotechnology, code no. sc‐32293, 1 : 1000 dilution). Horseradish peroxidase (HRP)‐conjugated secondary antibodies were used as follows: anti‐mouse IgG polyclonal (Medical & Biological Laboratories Co., Ltd., code no. 330, 1 : 2500 dilution) and anti‐rabbit IgG polyclonal (Medical & Biological Laboratories Co., Ltd., code no. 458, 1 : 2500 dilution).

### Cell cycle analysis

Cell cycle stages were analyzed on parental and transduced cells at passage 13 using a Muse Cell Analyzer (Millipore, Burlington, MA, USA) according to the manufacturer's instructions.

### F‐actin staining

All transduced cells and human fibroblast‐like cells derived from embryo (HE16 cells) (purchased from RIKEN, BioResource Center in March 2014) were cultured in a glass‐bottom CELLview cell culture dish (Greiner Bio‐One Co., Tokyo, Japan). Cultured cells were washed with PBS, fixed with 4% paraformaldehyde, and treated with 0.1% Triton X‐100 solution. Rhodamine‐X‐phalloidin solution (FUJI‐FILM Wako Pure Chemical Corporation) and Hoechst 33342 solution (Thermo Fisher Scientific) were used to detect F‐actin and nuclei in cultured cells. Fluorescence analysis was performed by a BZ‐X800 viewer (KEYENCE Co. Ltd., Osaka, Japan).

### Cellular senescence

Three types of parental cells at passage three and their transduced cells (K4DT and SV40) at over PD100 were seeded and grown up to 70–90% confluence. The culture cells were washed, fixed, and examined for the cellular senescence biomarker, namely senescence‐associated beta‐galactosidase (SA‐β‐Gal), using a senescence detection kit (BioVision, Milpitas, CA, USA) according to the manufacturer's instructions.

### Karyotype analysis

To examine the karyotypes of the cells, we cultured them in the presence of 150 ng·mL^−1^ of Colcemid solution overnight to promote cell metaphase. Cells were harvested, treated with 75 mm of KCl as a hypotonic solution, and fixed with a mixture of acetic acid and methanol. The treated cell samples were dropped onto a glass slide and stained with Giemsa solution. The karyotype was imaged using a microscope (KEYENCE), and the chromosomes were counted visually.

### Ethics statement

A dozen of bats, both males and females, were kept together in one exhibition area and allowed them to breed within group at the Osaka Municipal Tennoji Zoological Gardens in Japan. Therefore, it is difficult to determine when each individual was born and which parent is which. However, when staffs found a new baby (clearly identifiable as a sub‐adult), they captured it to insert a new chip (identification). One infant female bat, < 10 months old, had a relatively light body weight. Since confirmation occurred only after its death, there was no available individual information for this infant. The old female bat, born around autumn 2004, was identified on March 14, 2005. It had no notable medical history; however, it was dissected after its death to diagnosis pneumonia by veterinary doctor. Additionally, due to the absence of widespread occurrences, severe symptoms, or conditions notably different from known cases, routine bacterial testing was not carried out. Both individuals were born at the Osaka Municipal Tennoji Zoological Gardens, and their parents originated either from Tennoji or the Kochi Prefecture Zoo in Japan. The preceding generations of parents were likely born at either Tennoji or the Kochi Prefecture Zoo.

### Statistical analysis

The significance of cell cycle phase was analyzed using Tukey's multiple comparison procedure followed on the ANOVA analysis. Results with *P*‐values of < 0.05 were considered statistically significant and statistical significance was indicated by asterisks (*P < 0.05, **P < 0.01, and ***P < 0.001).

## Results

### Production of recombinant retrovirus and infection

**Fig. 1:**
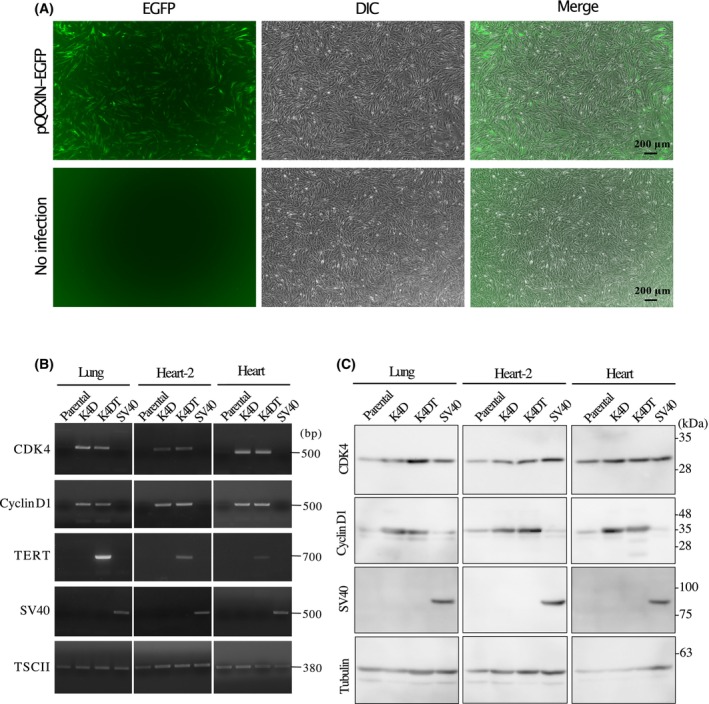
Detection of introduced genes in the cells derived from the Egyptian Rousettus bat. (A) Detection of EGFP expression in cells derived from the Egyptian Rousettus bat. Parental bat cells were exposed to EGFP‐expressing retrovirus (pQCXIN‐EGFP) for 96 h and EGFP expression was detected to confirm the efficiency of transgene infection. the scale bar is 200 μm. (B) Detection of introduced genes. Genomic DNA was extracted from the parental cells and their transduced cells and used to amplify the introduced genes. The endogenous TSCII gene was detected as an internal control gene. The band sizes (bp) are shown on the right side of the agarose gel images of the PCR detection. (C) Detection of protein expression of the introduced gene. The cell lysates of parental and transduced cells were prepared for the detection of protein expression of each introduced gene using its first detection antibody. Tubulin was detected as an internal control. The band sizes (kDa) are shown on the right side of the western blot images of protein detection.

We constructed retrovirus vector plasmids expressing mutant CDK4 (CDK4^R24C^), cyclin D1, TERT, and SV40 T antigens and transfected them with retrovirus packaging plasmids into 293T cells to produce retroviruses. Primary cells were isolated from the heart and lung tissues of two individual dead bats. Unfortunately, the lung fibroblasts from the 14 year old bat could not be successfully obtained due to contamination after death from pneumonia. Therefore, primary cells derived from the lung and hearts of an infant bat and the heart (designated as heart‐2) of an elderly bat were inoculated with the concentrated retroviruses, and the triply infected cells, which express CDK4^R24C^, cyclin D1, and TERT, were selected as described in the cell immortalization section of the Materials and methods. The resulting cells were named K4DT or SV40 according to the introduced genes. The infection efficiency of the transgenes was monitored based on EGFP expression using a fluorescence microscope (Fig. 1A).

### Detection of introduced gene and protein expression

To detect the transgene, genomic DNAs were extracted from parental and transduced cells and subjected to PCR. As shown in Fig. 1B, the expected sizes of the CDK4, cyclin D1, TERT, and SV40 genes were specifically detected in each type of transduced cell. For further confirmation, we examined the protein levels of the transgenes using western blotting. The expected molecular weights of the transgene products were determined using western blotting (Fig. 1C). Tubulin was used as the control. CDK4 and cyclin D1 bands were also detected in parental and SV40 cells, suggesting that the anti‐human CDK4 and anti‐human cyclin D1 antibodies cross‐reacted with the endogenous bat CDK4 and cyclin D1 proteins. CDK4 and cyclin D1 levels were higher in K4D and K4DT cells than in the parental cells. Interestingly, the CDK4 levels in SV40 cells were comparable to, or in the case of heart‐2 even higher, than those in K4D, and K4DT cells. The SV40 large T antigen band was specifically detected in SV40 cells. These results show that all transgenes were successfully transmitted into bat‐derived primary cells.

### Analysis of cell cycles

**Fig. 2:**
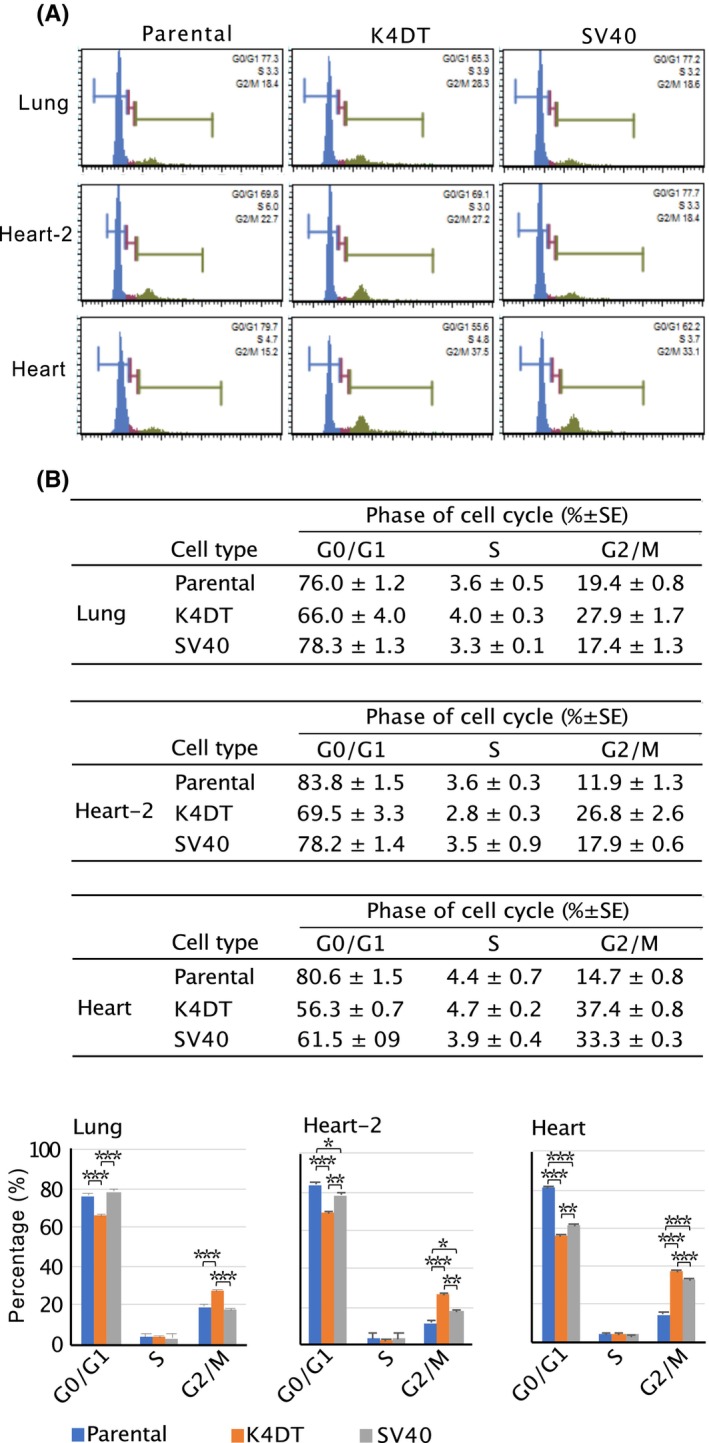
Analyses of the cell cycle in the Egyptian Rousettus bat‐derived cells. (A) Histogram analysis of the cell cycle in the parental and their transduced cells. Each cell type (1 × 10^5^) was harvested, fixed, and stained for cell cycle analysis. The representative detection data are shown. (B) Percentage of parental and transduced cells in each cell cycle phase. The mean ± standard error of the percentage of each cell cycle phase was shown. Each sample was performed in triplicate. Statistically significant differences were analyzed by Tukey's multiple comparison test and the *P*‐value was indicated by asterisks in the bar graphs (*P < 0.05, **P < 0.01, and ***P < 0.001).

Among the introduced genes, CDK4 and cyclin D1 are known to promote the G1 to S phase transition. Therefore, we used parental and transduced cells at passage 13 to analyze the cell cycle using a MUSE Cell Analyzer. Each cell type was tested in triplicate. Figure 2A shows representative cell cycle data for each type of parental, K4DT, and SV40 cells. The histogram shapes of the cell cycle data for each type of parental cell and their transduced cells showed similarities, with no major differences between the G1 and S phases. The average values of G0/G1, S, and G2 phases for each cell type are shown in Fig. 2B. Compared to their parental cells, K4DT cells showed a statistically significant decrease in the number of cells in the G0/G1 phase and a statistically significant increase in the number of cells in the G2/M phase. However, no significant differences were observed in the S phase between the parental and transduced cells. The results suggested that the G1 to S phase transition was promoted in all three types of K4DT cells, as well as in SV40 cells derived from the heart, such that their G2/M phase cells accumulated.

### Measurement of cell proliferation and cellular senescence in the transduced cells

**Fig. 3:**
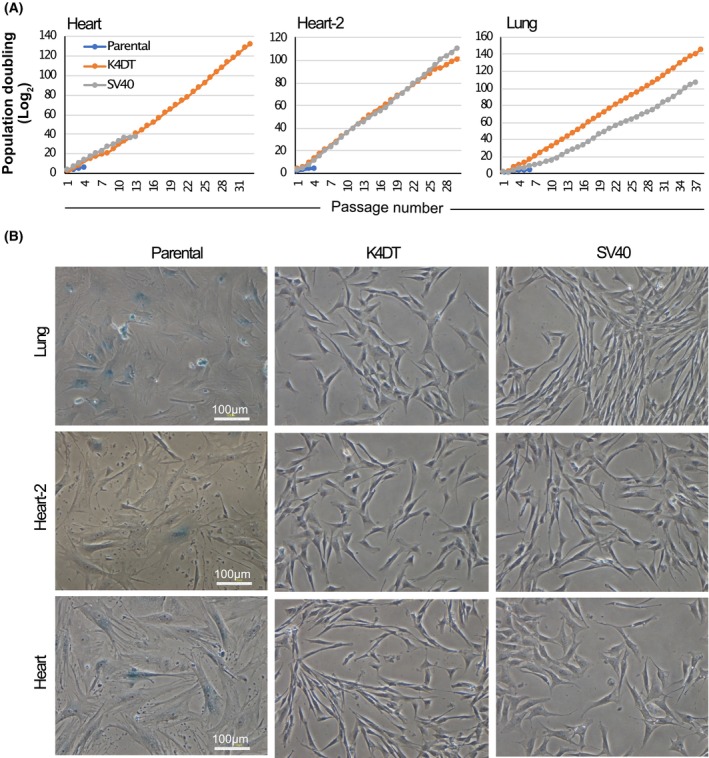
Cell proliferation and cell senescence in the Egyptian Rousettus bat‐derived cells. (A) Population doubling levels of parental and their transduced cells. Each cell type was seeded at an intensity of (5 × 10^4^ cells per well) on a 6‐well plate to incubate until a cell type reached 100% confluence and then collected for cell counting. The value of the cell division number was obtained by taking the ratio of the cell number at the 100% confluent point divided by the seeding cell number to log_2_. (B) Detection of cell senescence. A biomarker of cell senescence (senescence‐associated beta‐galactosidase) stained blue in all three types of parental cells. However, six types of transduced cells were negative for blue staining, indicating that they were not senescent. The scale bar is 100 μm.

The proliferative ability of transduced cells was also examined by sequential passages. Parental cells derived from the heart, heart‐2, and lungs had almost stopped proliferation at PDs of 13 (p4), 5 (p4), and 7.4 (p6), respectively (Fig. 3A). In contrast, the PDs of all transduced cells, except heart‐derived transduced SV40 cells, exceeded 100. A PD over 100 is considered as an arbitrary threshold line of cellular immortalization [28]. It was thought that the failure of heart‐derived transduced SV40 cells to divide to reach PD100 may be due to telomere shortening. We had also detected a cellular senescence biomarker, SA‐β‐Gal, and the result is shown in Fig. 3B. A typical senescent phenotype, enlarged cytoplasm, and blue‐stained SA‐β‐Gal were detected in all three types of parental cells at passage 3, but not in their transduced cells over PD100. This result indicated that the parental cells exhibited cellular senescence at an early stage (less than PD8), whereas the transduced cells remained free of cellular senescence above PD100. Based on these results, we concluded that five of the six types of transduced cells were immortalized.

### Karyotype of Egyptian Rousettus bat

**Fig. 4:**
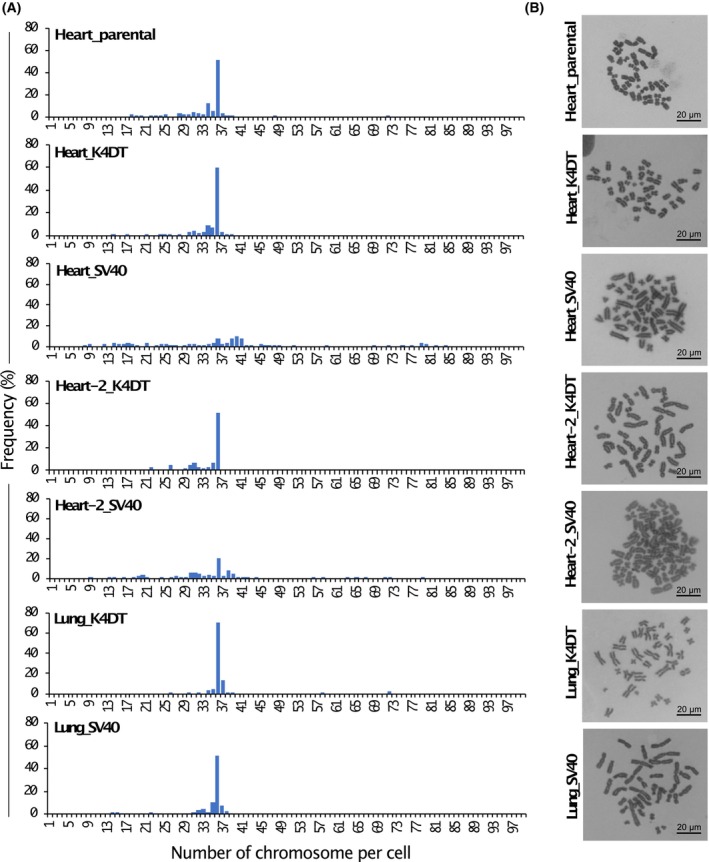
Karyotypes analysis on the parental and transduced cells. (A) Frequency of karyotypes. The metaphase of the cell cycle was promoted in the heart‐derived parental and six types of transduced cells and fixed for staining with a Giemsa solution. The karyotype of each cell type was imaged and manually counted in one hundred cells. Chromosome number frequencies were presented in histograms. (B) The representative detection of chromosome patterns on the heart‐derived parental cells and transduced cells. The scale bar is 20 μm.

In our previous study, K4DT cells derived from Ryukyu long‐furred rats showed karyotypes similar to their parental cells, whereas SV40 large T antigen‐transduced cells showed many aberrant chromosomes. SV40 cells lose characteristics of their original cells [29]. Therefore, we performed karyotype analysis using heart‐derived parental cells (p3) and transduced cells (above PD100) to clarify whether the introduced genes affect chromosomal instability. The chromosome number of Egyptian Rousettus bats was 36 for most of the 100 heart‐derived parental cell lines (Fig. 4A). Karyotype analysis could not be carried out successfully in the parental cells derived from bat heart‐2 and lungs due to cell division arrest. All three types of K4DT cells exhibited an apparently normal karyotype with 36 chromosomes. However, two of the heart‐derived SV40 cell lines showed numerous aneuploidies (Fig. S1). Unexpectedly, lung‐derived SV40 cells showed chromosomal patterns similar to those of K4DT cells, although we did not perform a precise karyotype analysis for translocations. Representative karyotype analyses of Egyptian Rousettus bats are shown in Fig. 4B. Egyptian Rousettus bats have 36 chromosomes, which is consistent with previously reported result [30].

### Distribution of F‐actin in transduced cells

**Fig. 5:**
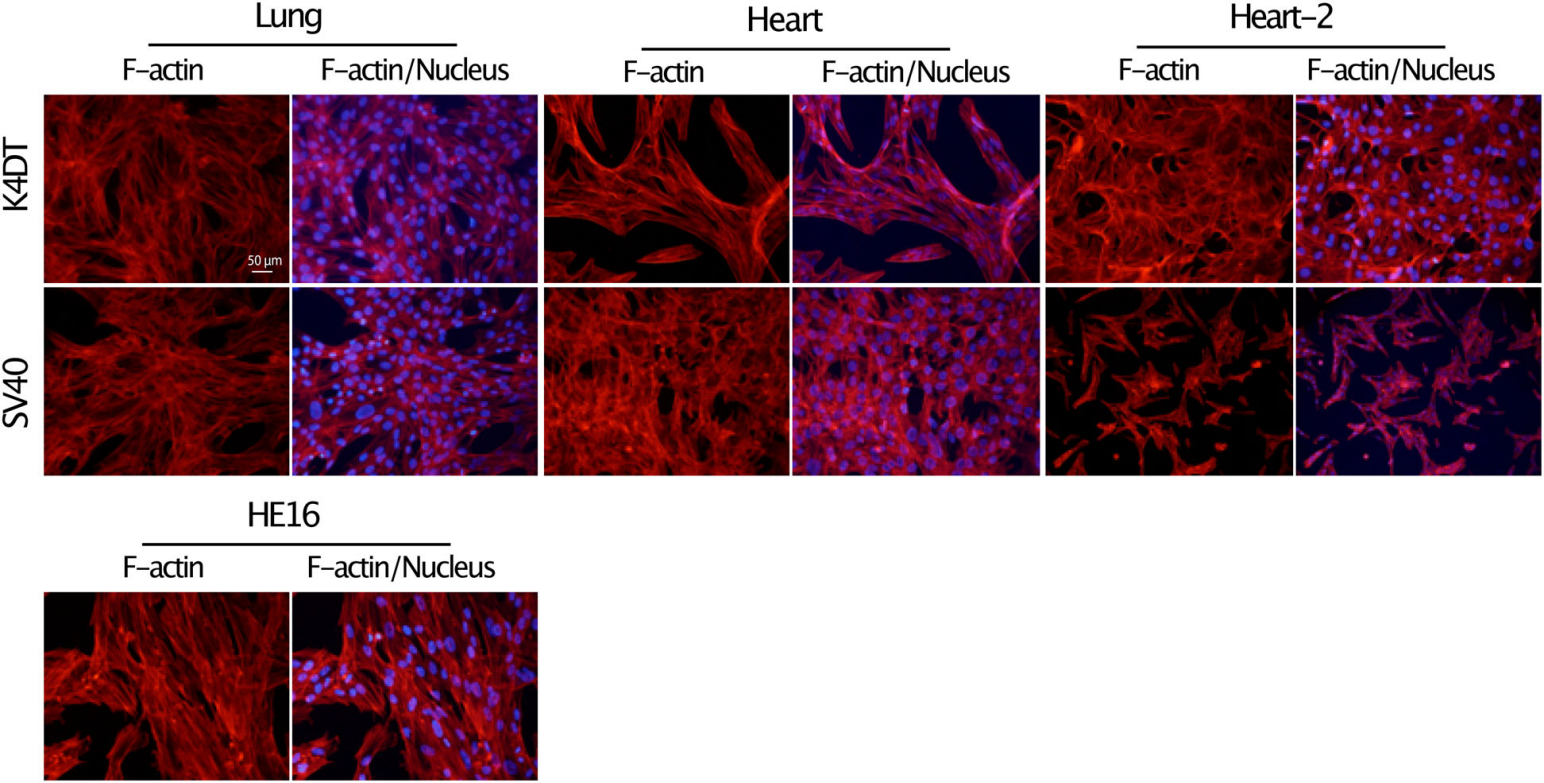
F‐actin distribution in transduced cells. All six types of transduced cells and Human fibroblast‐like cells derived from embryo (HE16 cells) were fixed on a glass‐bottom plate, and F‐actin and nuclei were stained with Rhodamine‐X‐phalloidin (red) and Hoechst 33342 (blue), respectively. The scale bar is 50 μm.

F‐actin plays a crucial role in the structure and function of the cytoskeleton, a dynamic network of protein filaments that provides structural support to the cell, helps maintain cell shape and is involved in various cellular processes. Therefore, in order to claim that these cells are fibroblast‐like, F‐actin was stained in transduced cells and HE16 cells. As shown in Fig. 5, the distribution of F‐actin in all transduced cells was similar to that in HE16 cells, with no significant differences. It was suggested that bat‐derived transduced cells are fibroblast‐like as well as HE16.

## Discussion

Previously, cells from Seba's short‐tailed bats and Straw‐colored fruit bats have been immortalized by expressing the SV40 large T antigen [31]. Brain, fetal, kidney, and lung cells from black flying foxes were immortalized by transferring the SV40 large T antigen. However, only a brain cell line was established by the expression of TERT alone, and other cell types could not be successfully established using TERT alone [32]. Further, other some bat cell lines were established by different immortalization methods (Table 2). The SV40 oncogene expression immortalization method resulted in karyotype alterations, whereas TERT expression did not induce genetic alterations, thus maintaining the parental cell characteristics [32]. Recently, we established Bonin flying box cell lines by expressing a combination of cell cycle regulators (mutant CDK4 and cyclin D1) and TERT, which closely resembled the cell cycle status of the original cells and showed minimal karyotypic changes [33]. Furthermore, fibroblasts have been identified as susceptible to various viruses, including respiratory syncytial virus, SARS‐CoV‐2, and influenza [34, 35, 36]. In the context of HIV‐I infection research, human fibroblast‐like cell lines play a crucial role in identifying potential target cells [37]. Consequently, fibroblast‐like cell lines are significant in viral infection studies, serving as important counterparts to epithelial cells. Moreover, it is a well‐established understanding that viruses affecting the respiratory system, especially the current pandemic strain SARS‐CoV‐2, possess the capability to cause both acute and prolonged chronic impairment in lung function [38]. Viral infection also causes inflammation of the heart muscle, which can lead to myocarditis [39, 40]. Therefore, we established fibroblast cell lines derived from the lungs and hearts of two individual bats by expressing either a combination of cell cycle regulators with TERT or SV40 large T antigen in this study.

**Table 2:** Some established bat cell lines and their basic information.

Bat type	Sex	Source	Organ	Immortalization method	Clone	References
Black flying fox	Female	Wild bat from Brisbane, Queensland	Bone marrow	hTERT	Failure	[32]
			Brain	SV40	PaBrT01‐03	
				hTERT	PaBrH01‐07	
			Foetus	SV40	PaFeT01‐10	
				hTERT	Failure	
			Foetal membranes	hTERT	Failure	
			Kidney	SV40	PaKiT01‐03	
				hTERT	Failure	
			Lung	SV40	PaLuT01‐04	
				hTERT	Failure	
			Testes	hTERT	Failure	
Bonin flying fox	Male	Wild bat from Chichijima, Japan	Skin	CDK4R24C, cyclin D1, hTERT	K4DT	[33]
European insectivorous bat	Unspecified		Kidney	Ebola virus envelope glycoprotein	MyDauLu/47.1	[57]
Pipistrellus ceylonicus bat	Female	India	Embryo		NOIV‐BtEPC	[58]
Tadaria brasiliensis	Female	Canada	Lung epithelial cells		Tb1Lu	[59]
Big brown bat	Male	Canada	Kidney	Myotis polyomavirus large T‐antigen	MyPVTag	[60]
				SV40	Failure	
Egyptian rousette	Unspecified	The Wilhelma Zoo in Stuttgart, Germany	Body of fetus	Adenovirus serotype 5 E1A	R06E	[8]
			Head of fetus	Adenovirus serotype 5 E1A	R05T	
			Vertebrate column of fetus	Adenovirus serotype 5 E1A	R05R	
	Female	Infant bat from the Osaka Municipal Tennoji Zoological Gardens, Japan	Lung	CDK4R24C, cyclin D1, hTERT	Lung_K4DT	Current study
				SV40	Lung_SV40	
			Heart	CDK4R24C, cyclin D1, hTERT	Heart_K4DT	
				SV40	Heart_SV40	
	Female	Adult bat from the Osaka Municipal Tennoji Zoological Gardens, Japan	Heart	CDK4R24C, cyclin D1, hTERT	Heart‐2_K4DT	
				SV40	Heart‐2_SV40	
	Unspecified		Kidney	Ebola virus envelope glycoprotein	RoNi/7.1	[57]

Most of the TERT sequence was conserved between humans and bats. However, 91 amino acids were destructed in bat TERT compared to human TERT based on the database (data not shown). Until now, the location of the TERT gene on bat chromosomes has not been readily available in the database. We do not determine the integration site of TERT in the bat chromosome in this study. According to the retrovirus studies [41, 42, 43], the retrovirus genes integrate randomly into the host cell DNA genome at an early stage and are removed/deleted by the DNA repair function and immunity. As a result, the number of polyclonal integration sites decreases. Thus, TERT was thought to be randomly integrated into the genome of the transduced bat cells. Furthermore, the mechanism by which the TERT gene is integrated into other chromosomes in a way that would directly affect its ability to prolong or reduce cell senescence through telomerase shortening is unknown. TERT plays a critical role in maintaining telomeres, the protective caps at the ends of chromosomes, and preventing cellular senescence.

Immortal cell lines, including tumor cell lines, maintain telomere length either by telomerase activation or through alternative lengthening of telomere (ALT) mechanisms [44, 45]. The SV40 T antigen extends the lifespan of primary animal cells by inactivating pRB and p53. However, because it cannot activate telomerase, most cell lines established by the transduction of the SV40 large T antigen have been reported to maintain their telomere length through ALT, which can be induced by the loss of ATRX function in most cases [44]. The SV40 large T antigen can induce a finite number of additional cell divisions beyond the point at which short telomere ends are exposed, which are recognized as double‐strand breaks. However, cells cease to proliferate and undergo a crisis, during which massive breakage‐fusion cycles produce aberrant chromosomes. In human cells, approximately 1 in 10^7^ fibroblasts and 1 in 10^5^ epithelial cells escape crisis through spontaneous activation of telomerase or ALT, and the cell culture becomes immortal [44]. Therefore, it was suggested that heart‐derived SV40 cells, which ceased proliferation at around 40 PDs due to the ATRX/DAXX chromatin remodeling complex with one or more as‐yet‐unidentified genetic or epigenetic alterations, failed to activate telomerase or ALT. Meanwhile, the heart‐2‐derived SV40 cells acquired active telomerase or ALT to escape the crisis, although only a slight growth retardation was observed between 40 and 60 PDs.

*Myotis myotis* is the longest living bat [46], with a maximum lifespan of 37.1 years, and does not express telomerase in its blood or fibroblasts. Ataxia‐telangiectasia mutated and senataxin are thought to mediate telomere dynamics during the repair and prevention of DNA damage in *Myotis* bats. However, telomere shortening with age has been observed in *Rhinolophus ferrumequinum* and *Miniopterus schreibersii*, which have maximum lifespans of 30.5 and 22 years, respectively [47]. The average lifespan of Egyptian Rosettes in the wild is 8–10 years, whereas in captivity, it is approximately 25 years [48]. In this study, primary cells were taken from 10 month old and 14 year old Egyptian Rosettes bats. Therefore, it was assumed that telomere shortening occurred in the cells of the Egyptian Rosette bats [48, 49].

The K4DT cells retained karyotypes similar to those of the original cells, whereas the SV40 cells derived from heart and heart‐2 showed abnormal karyotypes, such as deletions, disruptions, shifts, and eliminations (Fig. S1). These results are consistent with previous studies [29]. Additionally, immortalized SV40 cells derived from embryonic fibroblasts induce DNA damage at the G2/M checkpoint [50]. It was assumed that aberrant karyotypes were induced by continuous DNA damage in heart and heart‐2‐derived SV40 cells. Unexpectedly, 51% of lung‐derived SV40 cells showed similar chromosomal numbers to the original cells. Seventy‐four percent of giant panda skin fibroblast cell lines immortalized with the SV40 oncogene had a normal karyotype, as that of the original cells [51]. In this study, numerical chromosomal abnormalities were formally examined using karyotype analysis (Table 1). These results suggest that immortalization of bat cells with cell cycle regulators and TERT is superior to that with the SV40 T antigen in terms of chromosomal stability and retention of parental cell characteristics. Further characterization is required to determine whether they retain their normal diploid chromosomes in the future.

In our western blots, the same mobility bands corresponding to human CDK4 and cyclin D1 were detected in parental and SV40 cells. Because these genes are well‐conserved between humans and other animals, they are likely to be detected due to cross‐reactivity with antibodies, as previously shown in other animal cells [20, 52]. The successful immortalization of bat cells with three human genes (CDK4^R24C^, cyclin D1, and TERT) also suggests that their functions are conserved in humans and other animals, including bats [33, 53, 54, 55, 56]. This suggests that the K4DT method used herein is applicable to most primary animal cells to obtain immortalized cells that maintain their original characteristics and chromosomal stability.

## Conclusion

We established six Egyptian Rousettus bat‐derived cell lines by expressing human CDK4^R24C^, cyclin D1, TERT, and the SV40 large T antigen. All six types of transduced cells extended their lifespan and exhibited cell cycles similar to those of the early passage parental cells. All K4DT‐ and lung‐derived transduced SV40 cells had normal chromosome numbers (2n = 36). The other two types of SV40 cells showed many abnormal karyotypes, indicating that these SV40 cells have lose their original cellular characteristics. These established cell lines, particularly K4DT cells, are valuable research materials for studying pathogens. In the future, these cell lines may be promising for analyzing immune differences between reservoir hosts and humans or other animals.

## Conflict of interest

The authors declare no conflict of interest.

### Peer review

The peer review history for this article is available at https://www.webofscience.com/api/gateway/wos/peer‐review/10.1002/2211‐5463.13781.

## Author contributions

LB, TK (Tohru Kiyono), and TF conceived, designed, and wrote the manuscript. LB, TT, TK (Takeshi Kobayashi), RN, YK, YS, KT, HT, ES, TO, TK (Tohru Kiyono), and TF contributed to data validation and data analysis. All authors read and approved the final manuscript.

## Supporting information


**Fig. S1.** Karyotype analysis of heart‐derived SV40 and heart‐2‐derived SV40 cells. The representative data of the karyotype analysis such as deletions, disruptions, shifts, and eliminations of SV40 cells were presented (white arrows). Heart‐derived SV40 cells (top); Heart‐2‐derived SV40 cells (bottom). The scale bar is 50 μm.

## Data Availability

All data generated or analyzed for this study was included in this article. Our established cell lines have not been deposited elsewhere as of now. While we currently have no intentions of commercializing these cells, we are open to providing them to researchers engaged in non‐commercial scientific research, thereby fostering collaboration and contributing to the advancement of scientific knowledge in this field.
