# Supplementary material for: Establishment of immortalized Egyptian Rousettus bat cell lines
Source: FEBS Open Bio. 2024 Feb 19;14(4):598–612. doi: 10.1002/2211-5463.13781 (PMC10988675; doi:10.1002/2211-5463.13781)
Supplement: Supplementary file 1 — Fig. S1. Karyotype analysis of heart‐derived SV40 and heart‐2‐derived SV40 cells. The representative data of the karyotype analysis such as deletions, disruptions, shifts, and eliminations of SV40 cells were presented (white arrows). Heart‐derived SV40 cells (top); Heart‐2‐derived SV40 cells (bottom). The scale bar is 50 μm. [file FEB4-14-598-s001.pdf]

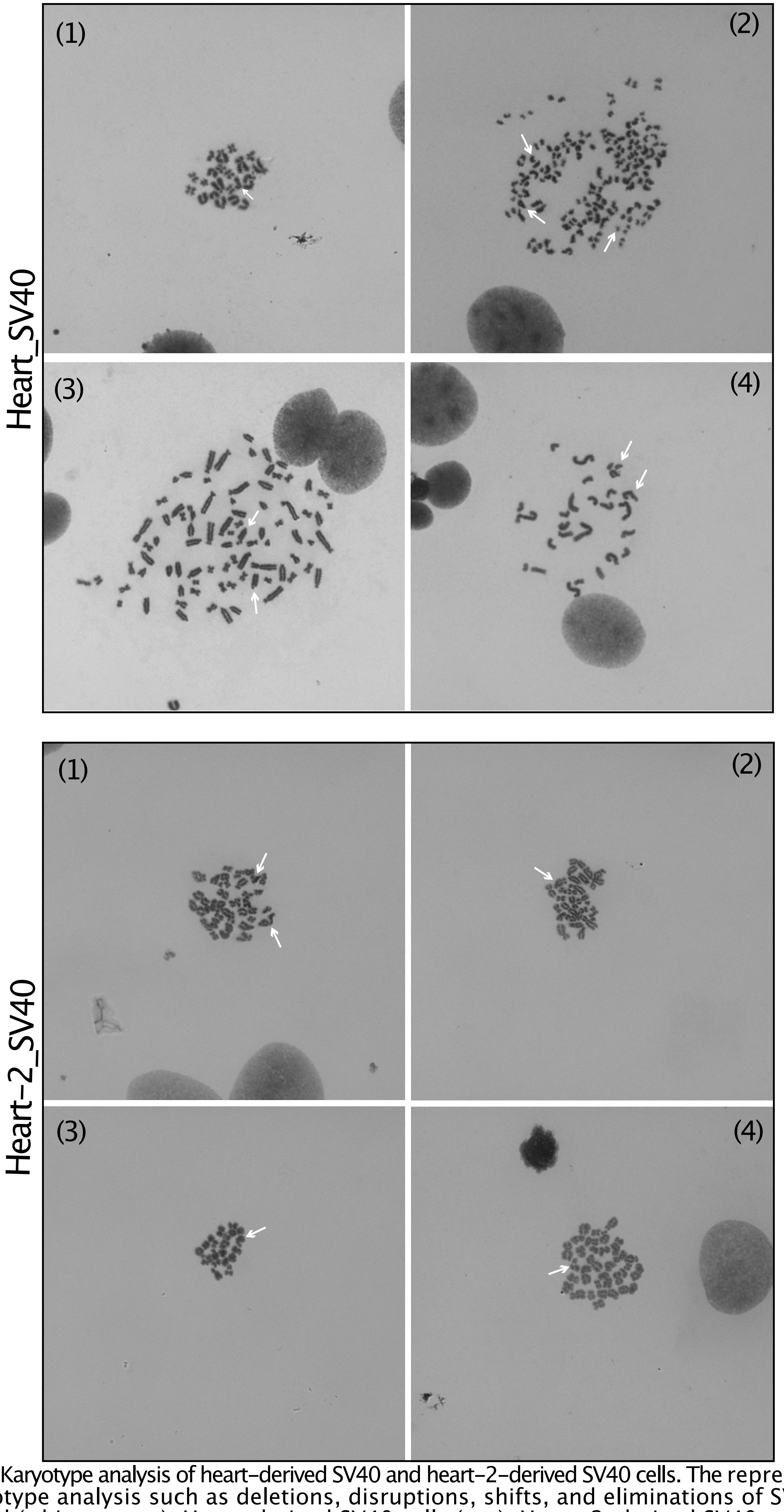

Figure S1. Karyotype analysis of heart-derived SV40 and heart-2-derived SV40 cells. The representative data of the karyotype analysis such as deletions, disruptions, shifts, and eliminations of SV40 cells were presented (white arrows). Heart-derived SV40 cells (top); Heart-2-derived SV40 cells (bottom).
